# Prognosis-based management of unexplained infertility—why not?

**DOI:** 10.1093/hropen/hoae015

**Published:** 2024-03-22

**Authors:** Laxmi Shingshetty, Rui Wang, Qian Feng, Abha Maheshwari, Ben W Mol

**Affiliations:** Aberdeen Centre for Women's Health Research, Institute of Applied Health Sciences, School of Medicine, Medical Sciences and Nutrition, University of Aberdeen, Aberdeen, UK; Department of Reproductive Medicine, NHS Grampian, Aberdeen, UK; Department of Obstetrics and Gynaecology, Monash University, Clayton, VIC, Australia; Department of Obstetrics and Gynaecology, Monash University, Clayton, VIC, Australia; Aberdeen Centre for Women's Health Research, Institute of Applied Health Sciences, School of Medicine, Medical Sciences and Nutrition, University of Aberdeen, Aberdeen, UK; Department of Reproductive Medicine, NHS Grampian, Aberdeen, UK; Aberdeen Centre for Women's Health Research, Institute of Applied Health Sciences, School of Medicine, Medical Sciences and Nutrition, University of Aberdeen, Aberdeen, UK; Department of Obstetrics and Gynaecology, Monash University, Clayton, VIC, Australia

**Keywords:** unexplained infertility, prognostic models, prediction models, expectant management, intra-uterine insemination, *in vitro* fertilization, live birth

## Abstract

Up to a half of couples seeking medical assistance for infertility are diagnosed with unexplained infertility, characterized by normal ovulation, tubal patency, and semen analysis results. This condition presents a challenge in determining the optimal treatment approach. Available treatments include IUI and IVF, but guidelines vary on when to offer each. Prognosis-based management is identified as a research priority, and various prediction models have been developed to guide treatment decisions. Prognostic factors include female age, duration of subfertility, and sperm parameters, among others. Prognosis-based strategies can enhance cost-effectiveness, safety, and patient outcomes, offering less invasive options to those with good prognoses and more aggressive interventions to those with poor prognoses. However, there is a gap between research evidence and its clinical application. In this article, we discuss the application of prognosis-based management in the context of unexplained infertility, highlighting its potential to improve clinical decision-making and patient outcomes.

## Introduction

In up to half of all couples who seek medical assistance for infertility, a direct cause for their inability to conceive cannot be identified. They are therefore labelled as having unexplained infertility (Buckett and Sierra, 2019). Unexplained infertility is characterized by normal ovulation and at least one patent Fallopian tube in the female partner, as well as a normal (or slightly deviant) semen analysis in the male partner according to the World Health Organization criteria (Cooper *et al.*, 2010). The causes of unexplained infertility remain, as determined by its definition, largely unknown (Quaas and Dokras, 2008). While couples with unexplained infertility even after 12 months of trying still have inherent chances of conception, they are typically considered to be less fertile. They may therefore, despite the lack of a clear understanding of the underlying physiological factors responsible for their reduced fertility, undergo a variety of diagnostic and therapeutic procedures (Wang *et al.*, 2020; Raperport *et al.*, 2023). On one hand, lack of conception might be put down to chance, whereas on the other hand, underlying unidentified and maybe even unknown causes for infertility prevent conception.

Available medical treatments for unexplained infertility include IUI with ovarian hyperstimulation and IVF. Treatments such as IUI and IVF might on one hand be effective because they increase the number of possible conceptions, for example by creating multiple follicles or embryos, but they also might overcome an unknown barrier to conception.

At present, the consensus on the management of unexplained infertility across continents is varied. For example, The National Institute for Health Care and Excellence (NICE) guideline (National Institute for Health and Care Excellence, 2013) for unexplained infertility recommends IVF after 2 years of expectant management. The American Society of Reproductive Medicine (ASRM) and the Canadian Fertility and Andrology Society recommend an initial approach of IUI with superovulation for up to three to four cycles followed by IVF, but the true challenge lies in determining the optimal timing for each treatment (Buckett and Sierra, 2019; Penzias *et al.*, 2021). A recent ESHRE guideline also discusses the management of unexplained infertility and recommends IUI with ovarian stimulation as a first-line treatment for couples with unexplained infertility (Teede *et al.*, 2018; Romualdi *et al.*, 2023).

In couples diagnosed with unexplained infertility, natural conception is inherently a possibility. Therefore, assessing the prognostic profile of these couples becomes crucial in understanding the likelihood of natural conception. This opportunity for prognosis-based management of unexplained infertility has been identified as one of the top 10 priorities for infertility research (Duffy *et al.*, 2020); however, it was not used in the above-mentioned guidelines to phrase the key research questions. The ESHRE guideline does indeed recognize the potential of a prognosis-centred strategy for couples facing unexplained infertility. However, it seems to adopt a more generalized stance, placing the onus on readers and clinicians to select their preferred methodologies (Romualdi *et al.*, 2023).

Here, we discuss the indication-setting for the treatment of couples with unexplained infertility. A first and fundamental step prior to the management of these couples is the exclusion of diagnoses linked to an underlying cause of infertility. We will then demonstrate how prognosis can be used to determine which couples require treatment and at what stage of their infertility.

## Unexplained infertility is a diagnosis per exclusion

The main diagnoses linked to a cause of infertility are anovulation, severe male factor, or tubal obstruction. There should be an egg, there should be sperm and the two should come together. If any of these three factors is missing, conception will not occur and infertility is a fact.

The diagnosis of anovulation, or its opposite, the confirmation of ovulation can be made through a variety of methods such as urinary LH tests, serum progesterone levels, basal body temperature charting, and ultrasound (Romualdi *et al.*, 2023). The first-line and second-line treatments of an- or oligo-ovulation are medical ovulation induction (Teede *et al.*, 2018). When this is not successful, IVF and/or laparoscopy with ovarian drilling can be applied.

Male infertility is diagnosed or excluded by conducting a simple semen analysis (The Sixth Edition of WHO Manual for Semen Analysis; Boitrelle *et al.*, 2021). Severe male factor infertility involves severe oligozoospermia (<5 × 10^6^ sperm per ml of ejaculate), asthenozoospermia, or even azoospermia (Mazzilli *et al.*, 2022; Sharlip *et al.*, 2002). Its treatment requires IVF and ICSI. In men with azoospermia, testicular sperm extraction or treatment with donor sperm can be considered. Finally, tubal blockage, or its opposite, namely patency of both or at least one Fallopian tube, can be diagnosed with hysterosalpingography and laparoscopy, and more recently hystero-salpingo contrast sonography (Penzias *et al.*, 2021). Treatment could be tubal surgery, but is in most settings dominated by IVF.

Couples diagnosed with anovulation, severe oligozoospermia or azoospermia, and double-sided tubal blockage have one similarity: without treatment, their chances to conceive are—close to nil—treatment is therefore indicated in these couples (Thurston *et al.*, 2019).

A special category is female ageing. Since ovarian reserve decreases with age, fertility declines too (Amanvermez and Tosun, 2016). In other words, even without any abnormalities, female fertility declines, with a turning point somewhere between 35 and 40 years. Above that turning point, infertility can be related to reduced ovarian competency and the potential benefits of ART are unlikely if infertility is attributed to the aging of the woman.

While there are many more diagnoses possible, the above category covers the majority of the cases where a diagnosis can be made. The remaining couples are diagnosed with unexplained infertility, and these couples do, by definition, not have a fundamental impediment to conception (Quaas and Dokras, 2008). As a consequence, natural conception is expected to occur in a substantial number of these women. As it is not useful to treat couples who have good prospects of natural conception in due time, before deciding on the start of treatment, a thorough understanding of the prognosis for natural conception is therefore needed. If the prognosis for a ‘natural pregnancy’ in due course is high enough, it might be justified to delay treatment in these couples for some time.

## Prognosis and unexplained infertility

The adoption of a prognosis-based approach has been steadily increasing in the field of medicine, proving to offer significant advantages for healthcare systems and patients alike. This approach is evident across various medical domains, such as oncology, where tools like the Nottingham Prognostic Index and Adjuvant Online have been developed to predict the prognosis of young breast cancer patients (<40 years old) (Hearne *et al.*, 2015). Similarly, there is a growing demand for the implementation of a prognosis-based strategy in the management of couples facing unexplained infertility. This entails an initial assessment of the overall prognosis for untreated couples and an examination of the factors influencing this prognosis. Subsequently, these influential factors are integrated into a predictive model to distinguish between couples with a reasonable likelihood of natural conception and those who may require intervention to facilitate conception. Such a methodological framework aligns with the PROGRESS Framework, and we emphasize the importance of adhering to these recommendations when managing couples dealing with unexplained subfertility (Hemingway *et al.*, 2013).

Prognostic research in reproductive medicine was initiated 30 years ago, when Bosofte suggested the period of infertility, the female infertility factor, and sperm penetration as prognostic factors (Bostofte *et al.*, 1993). Since then, several prediction models have been designed for natural conception (Hunault *et al.*, 2004; Bensdorp *et al.*, 2017; van Eekelen *et al.*, 2017; McLernon *et al.*, 2019), IUI (Tomlinson *et al.*, 1996; Steures *et al.*, 2004; Erdem *et al.*, 2008; Souter *et al.*, 2022), and IVF (Hughes *et al.*, 1989; Nayudu *et al.*, 1989; Stolwijk *et al.*, 1996; Templeton *et al.*, 1996; Tomlinson *et al.*, 1996b; Bancsi *et al.*, 2000; Smeenk *et al.*, 2000; Ferlitsch *et al.*, 2004; Steures *et al.*, 2004; Loendersloot *et al.*, 2011; Nelson and Lawlor, 2011; Dhillon *et al.*, 2016; McLernon *et al.*, 2016; Souter *et al.*, 2022). Table 1 describes different prediction models and the model stages.

**Table 1.** Prediction models for pregnancy after expectant management, IUI, and IVF.

	Model	**Sample size** ^a^	End point	Predictive factors	Performance	Model stage
**Natural conception**	Hunault *et al.*, 2004	2459	Live birth	Duration of subfertility, female age, pregnancy history, sperm factor, and post-coital test	Reliability ratio (calibration): 0.8–1.2	Validation
	Bensdorp *et al.*, 2017	5184	Live birth	Hunault, 2004 model plus female BMI, cycle length, basal FSH levels, tubal status, history of previous pregnancies, semen volume, and morphology	Calibration: 0.71 Net reclassification index 0.14	Validation
	van Eekelen *et al.*, 2017	4999	Ongoing pregnancy	Female age, duration of subfertility, primary or secondary subfertility, percentage of motile sperm, and referral type	Calibration plot showed fair calibration	Validation
	McLernon *et al.*, 2019	1316	Live birth	Female age, duration of infertility, previous pregnancy status, and year of registrations predictors	Calibration slop 0.968	Validation

**IUI**	Steures *et al.*, 2004	3371 couples with 14 968 cycles	Ongoing pregnancy	Female age, duration of subfertility, type of diagnosis, ovarian hyperstimulation, and cycle numbers	The difference between observed and expected outcomes is <0.5% (calibration)	Validation

**IVF**	Templeton *et al.*, 1996	36 961 cycles	Live birth	Female age, duration of infertility, previous live birth, female causes of infertility, and number of previous unsuccessful IVF	3 studies showed poor calibration and 1 showed good calibration	Model derivation
	Nelson and Lawlor, 2011	144 018 cycles	Live birth	Female age, duration of subfertility, previous unsuccessful IVF, and the use of own oocytes	3 studies have shown excellent calibration	Validation
	Loendersloot *et al.*, 2011	2621 cycles	Ongoing pregnancy	13 variables in final model	2 validation studies have shown fair calibration	Validation
	Dhillon *et al.*, 2016	9915 cycles	Live birth	Female BMI, ovarian reserve, and ethnicity	1 validation study showing near perfect calibration	Validation
	McLernon *et al.*, 2016	113 873	Live birth	Pre-treatment model: female age and duration of infertility. Post-treatment model: female age, number of eggs collected, and cryopreservation of embryos	Optimal calibration as per 1 validation study	Validation

If not specified, the unit is the number of couples.

From the models above, the Hunault model integrated data from three previous cohorts into a unified prediction model, and found female age, duration of subfertility, primary or secondary subfertility, percentage of motile sperm, and referral status (being referred by a general practitioner or a fellow gynaecologist) to be independently predictive for natural conception (Hunault *et al.*, 2004).

Before prognostic models can be used in clinical practice, they need to be validated in cohort studies or, even better, their impact should be assessed in randomized controlled trials (RCTs) (Leushuis *et al.*, 2009). The Hunault model demonstrated good calibration during externally validated studies in the Netherlands, New Zealand, and Australia (van der Steeg *et al.*, 2007; Farquhar *et al.*, 2011; Song *et al.*, 2021). Bensdorp *et al.* (2015) extended the model with additional variables such as cycle length, BMI of the woman, a basal FSH level, and a semen analysis.

More recently, updated prediction models (van Eekelen *et al.*, 2017; McLernon *et al.*, 2019) have been designed that provide predictions over time that allow adjustments at a repeated interval of time that could assist in offering chances of conception over multiple time intervals and has a distinct advantage of being able to provide prediction once the couple has returned after initial expectant management. At their return after the initial expectant management, the estimates of conception can be recalculated using one of the dynamic prediction models (van Eekelen *et al.*, 2017; McLernon *et al.*, 2019). While these ‘dynamic’ prediction models foresee a need for personalized predictions, they are not needed in a guideline, where prediction models should guide not more than the first decision to start or delay treatment.

The prediction models can be used to determine if IUI and IVF are expected to add to natural fertility chances. It should be realized that in different situations, many predictive factors will work in the same direction and the same magnitude. For example, increased female age will reduce the fertility chances, independent of whether these are natural pregnancy chances or success after IUI or IVF. Predictors that strongly decrease natural fertility chances, but do not or to a lesser extent reduce fertility chances after treatment with IUI and IVF, are more suitable treatment selection markers. Examples of these are the duration of infertility, but maybe also the prognostic index derived from the Hunault model or other prediction models.

**Figure 1.**
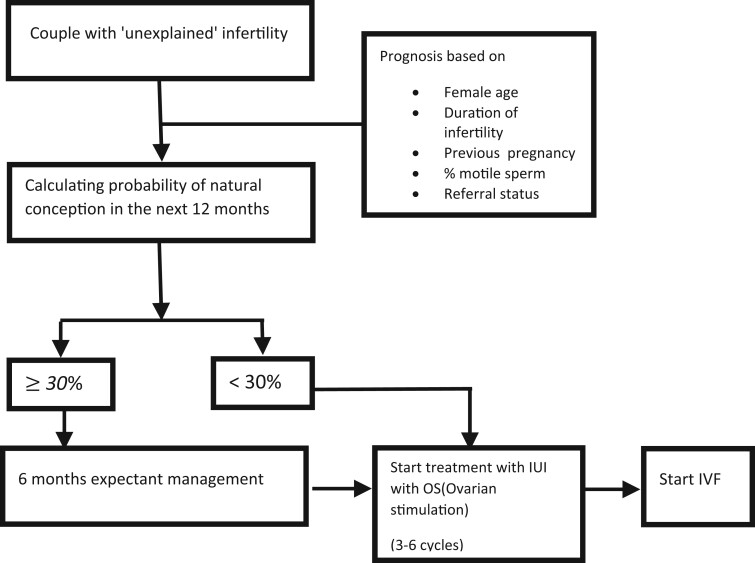
**Management strategies for couples with unexplained infertility**. Note: After exclusion of other causes, couples are assessed by calculating a prognostic index for natural conception. In case of a favourable prognosis (natural conception chances with 12 months >30%) expectant management for 6 months is the preferred strategy. In other couples, intrauterine insemination with ovarian stimulation for three to six cycles should be the preferred strategy.

Prediction models can be used to triage couples with unexplained infertility as having a good, intermediate, or poor prognosis for natural conception. The power of using the prediction models lies in their capacity to guide treatments (Fig. 1). Couples with good prospects for natural conception—say >30% conception chances in 12 months—can initially be managed expectantly, while in couples with poor prognosis for natural conception—<30% conception chances in 12 months—but good prognosis after treatment, IUI, and later IVF/ICSI are justified (Steures *et al.*, 2006). In couples with poor prognosis for natural conception where treatment is not expected to increase these chances, for example owing to a higher female age, IVF with oocyte donation has the best chance to overcome the low natural conception chances.

Importantly, the use of prediction models has not only been validated in cohort studies but also their impact has been assessed in RCTs. Steures *et al.* (2006) randomized 253 couples with an intermediate prognosis for natural conception (30–40% in 12 months) and found that IUI with hyperstimulation did not increase ongoing pregnancy rates after 6 months. In contrast, two other RCTs, the TUI (The Uterine Insemination) and the EXIUI (Expectant management vs IUI in unexplained subfertility and a poor pregnancy prognosis) trials, were carried out in couples with a poor prognosis for natural conception (<30% in 12 months) (Farquhar *et al.*, 2018; Wessel *et al.*, 2022). These trials, both with a large sample size, clearly demonstrated that IUI with ovarian stimulation is more effective than expectant management in these women. Bensdorp *et al.* (2015) showed in a large RCT that in these couples IVF does not bring a benefit over IUI with ovarian stimulation.

Thus, the prognostic index for natural conception, as established with the Hunault model, can be used as a treatment selection marker in couples with unexplained infertility (Hunault *et al.*, 2004). In couples with a good prognosis, IUI with ovarian stimulation does not benefit over expectant management, while in poor prognosis couples, IUI with hyperstimulation should be recommended, with cancellation policies in the presence of multiple follicular development to reduce the risk of multiple pregnancy (Wang *et al.*, 2019b; Wessel *et al.*, 2022).

Several other ingredients can be added to the management plan. Lifestyle interventions should be considered where appropriate. Also, tubal patency testing with oil-based contrast is known to increase live birth rates in couples with unexplained infertility, in couples with a good prognosis (Wang *et al.*, 2019a; Zhang *et al.*, 2022).

## Prediction models and their adoption on a wider scale

Prediction models have been in existence for over three decades, yet their widespread integration into routine practice remains limited. Factors contributing to this underutilization include a lack of awareness about these models, concerns regarding their trustworthiness and appropriateness for practical use, and insufficient validation across diverse populations. In the context of fertility evaluation, couples often opt for immediate interventions over natural conception because of the perceived need for ‘treatment’ and impatience regarding the time required for natural conception.

The successful adoption of prediction models hinges on robust validation efforts in diverse populations, ensuring the models’ generalizability and reliability. A critical aspect of achieving broader implementation involves raising awareness among clinicians about the clinical utility of prognostic models. Demonstrating how the incorporation of these models can lead to enhanced patient outcomes, improved decision-making, and resource optimization is essential. Moreover, the design of prognostic models should prioritize accessibility and user-friendliness for healthcare professionals, with careful consideration given to their seamless integration into existing healthcare systems (Collins *et al.*, 2024).

While certain models exist, the Hunault model demonstrated good calibration during externally validated studies, making it a more generalizable and reliable model (Custers *et al.*, 2007; van der Steeg *et al.*, 2007). In spite of its validation on a wider scale, local application and validation are recommended before using in routine practice to improve the accuracy of the model performance. Alternative models, such as van Eekelen’s and McLernon’s (van Eekelen *et al.*, 2017; McLernon *et al.*, 2019), although existing, require further validation, especially in diverse populations. Practitioners in different geographical locations can effectively leverage prediction models by comprehensively understanding their purpose, clinical relevance, and underlying predictors. Regularly auditing model performance in conjunction with clinical judgment is essential for continued efficacy. Addressing the inclination towards immediate interventions requires patient education to empower informed decision-making between expectant routes and more intensive procedures such as IUI or IVF. A prospective survey exploring barriers and future adoption trends of these models would provide valuable insights for refining clinical practice.

In the endeavour to increase awareness and promote the adoption of prediction models, international societies can play a pivotal role. The active engagement of these models on international platforms, such as ESHRE or ASRM, can serve as a catalyst in illuminating their existence. By featuring these models prominently within the discourse of international societies, a broader audience of clinicians can be reached, transforming them into advocates for the incorporation of prognosis-based approaches to unexplained infertility. This concerted effort would contribute to the realization of these models as a practical and beneficial tool in the clinical management of infertility.

## Advantages of prognosis-based management

Contemporary management of couples with unexplained infertility exhibits variation across different continents. In countries where reimbursement is available for assisted conception treatment, a pragmatic approach is adopted, wherein less invasive therapies are initially considered followed by more invasive interventions if the initial treatment proves unsuccessful. Conversely, in many countries where couples pay for infertility treatments entirely, or are largely out-of-pocket, IVF is offered as the first-line treatment despite its invasive nature, lower cost-effectiveness, and associated safety concerns.

The adoption of a prognosis-based approach confers several advantages, such as increased cost-effectiveness, improved safety, and reduced invasiveness or medicalization. Pham *et al.* (2018) found that if 90% of couples with a good prognosis delayed IVF treatment by 6 months, there would be a substantial decrease in the cost without compromising pregnancy and live birth rates over an 18-month period. Other studies have confirmed the cost-effectiveness of this approach (van Eekelen *et al.*, 2020; Tjon-Kon-Fat *et al.*, 2015). On top of that, adopting a prediction-based approach offers advantages in terms of providing couples with less invasive and safer options at an earlier stage of their infertility, while those with a poor prognosis can proceed directly to treatments such as IUI with ovarian stimulation and IVF. Even IVF with single embryo transfer carries an increased risk of twins or of multiple pregnancies (Chaabane *et al.*, 2015). On top of that, both IUI with ovarian stimulation and IVF bear an increased risk of perinatal complications such as preterm births, pre-eclampsia, and gestational diabetes (Pandey *et al.*, 2012).

Owing to the aforementioned considerations, it is in our opinion crucial to evaluate couples with unexplained infertility to assess the individual prognosis as part of the clinical management. The validity of this approach has been confirmed in multiple RCTs in which the prognostic index is used as an inclusion criterion, the strongest type of validation available.

## Authors’ roles

LS and BM conceived the initial idea for this commentary paper, developed the arguments within it, and drafted the manuscript. AM, RW, and QF critically revised the manuscript added additional information and references. All authors critically reviewed and edited the manuscript.

## References

[hoae015-B1] Amanvermez R , Tosun M. An update on ovarian aging and ovarian reserve tests. Int J Fertil Steril 2016;9:411–415. 26985328 10.22074/ijfs.2015.4591 PMC4793161

[hoae015-B2] Bancsi LF , Huijs AM, den Ouden CT, Broekmans FJ, Looman CW, Blankenstein MA, Te Velde ER. Basal follicle-stimulating hormone levels are of limited value in predicting ongoing pregnancy rates after in vitro fertilization. Fertil Steril 2000;73:552–557. 10689012 10.1016/s0015-0282(99)00552-x

[hoae015-B3] Bensdorp AJ , Steeg JWvd, Steures P, Habbema JDF, Hompes PGA, Bossuyt PMM, Veen FVD, Mol BWJ, Eijkemans MJC, Kasteren YMv; CECERM Study Group. A revised prediction model for natural conception. Reprod Biomed Online 2017;34:619–626. 28434653 10.1016/j.rbmo.2017.03.014

[hoae015-B4] Bensdorp AJ , Tjon-Kon-Fat RI, Bossuyt PMM, Koks CAM, Oosterhuis GJE, Hoek A, Hompes PGA, Broekmans FJM, Verhoeve HR, Bruin JPd et al Prevention of multiple pregnancies in couples with unexplained or mild male subfertility: randomised controlled trial of in vitro fertilisation with single embryo transfer or in vitro fertilisation in modified natural cycle compared with intrauterine insemination with controlled ovarian hyperstimulation. BMJ 2015;350:g7771. 25576320 10.1136/bmj.g7771 PMC4288434

[hoae015-B5] Boitrelle F , Shah R, Saleh R, Henkel R, Kandil H, Chung E, Vogiatzi P, Zini A, Arafa M, Agarwal A. The Sixth Edition of the WHO Manual for Human Semen Analysis: a critical review and SWOT analysis. Life 2021;11:1368. 34947899 10.3390/life11121368 PMC8706130

[hoae015-B6] Bostofte E , Bagger P, Michael A, Stakemann G. Fertility prognosis for infertile couples. Fertil Steril 1993;59:102–107. 8419195 10.1016/s0015-0282(16)55623-4

[hoae015-B7] Buckett W , Sierra S. The management of unexplained infertility: an evidence-based guideline from the Canadian Fertility and Andrology Society. Reprod Biomed Online 2019;39:633–640. 31439397 10.1016/j.rbmo.2019.05.023

[hoae015-B8] Chaabane S , Sheehy O, Monnier P, Bissonnette F, Trasler JM, Fraser W, Bérard A. Association between ovarian stimulators with or without intrauterine insemination, and assisted reproductive technologies on multiple births. Am J Obstet Gynecol 2015;213:511.e1–511.e14. 10.1016/j.ajog.2015.06.028 26079626

[hoae015-B9] Collins GS , Dhiman P, Ma J, Schlussel MM, Archer L, Calster BV, Harrell FE, Martin GP, Moons KGM, Smeden MV et al Evaluation of clinical prediction models (part 1): from development to external validation. BMJ 2024;384:e074819. 38191193 10.1136/bmj-2023-074819 PMC10772854

[hoae015-B18572694] Cooper TG, , Noonan E, , Von Eckardstein S, , Auger J, , Baker HWG, , Behre HM, , Haugen TB, , Kruger T, , Wang C, , Mbizvo MT et al World Health Organization reference values for human semen characteristics. Hum Reprod Update 2010;16:231–245. 19934213 10.1093/humupd/dmp048

[hoae015-B10] Custers IM , Steures P, Steeg JWvd, Dessel TV, Bernardus RE, Bourdrez P, Koks CAM, Riedijk WJ, Burggraaff JM, Veen FVD et al External validation of a prediction model for an ongoing pregnancy after intrauterine insemination. Fertil Steril 2007;88:425–431. 17408625 10.1016/j.fertnstert.2006.12.007

[hoae015-B11] Dhillon RK , McLernon DJ, Smith PP, Fishel S, Dowell K, Deeks JJ, Bhattacharya S, Coomarasamy A. Predicting the chance of live birth for women undergoing IVF: a novel pretreatment counselling tool. Hum Reprod 2016;31:84–92. 26498177 10.1093/humrep/dev268

[hoae015-B12] Duffy JMN , Adamson GD, Benson E, Bhattacharya S, Bhattacharya S, Bofill M, Brian K, Collura B, Curtis C, Evers JLH et al; Priority Setting Partnership for Infertility. Top 10 priorities for future infertility research: an international consensus development study. Hum Reprod 2020;35:2715–2724. 33252677 10.1093/humrep/deaa242 PMC7744161

[hoae015-B13] Erdem A , Erdem M, Atmaca S, Korucuoglu U, Karabacak O. Factors affecting live birth rate in intrauterine insemination cycles with recombinant gonadotrophin stimulation. Reprod Biomed Online 2008;17:199–206. 18681993 10.1016/s1472-6483(10)60195-2

[hoae015-B14] Farquhar CM , Boogaard NM, van den Riddell C, MacDonald A, Chan E, Mol BW. Accessing fertility treatment in New Zealand: a comparison of the clinical priority access criteria with a prediction model for couples with unexplained subfertility. Hum Reprod 2011;26:3037–3044. 21896547 10.1093/humrep/der279

[hoae015-B15] Farquhar CM , Liu E, Armstrong S, Arroll N, Lensen S, Brown J. Intrauterine insemination with ovarian stimulation versus expectant management for unexplained infertility (TUI): a pragmatic, open-label, randomised, controlled, two-centre trial. Lancet 2018;391:441–450. 29174128 10.1016/S0140-6736(17)32406-6

[hoae015-B16] Ferlitsch K , Sator MO, Gruber DM, Rücklinger E, Gruber CJ, Huber JC. Body mass index, follicle-stimulating hormone and their predictive value in in vitro fertilization. J Assist Reprod Genet 2004;21:431–436. 15704518 10.1007/s10815-004-8759-1 PMC3455618

[hoae015-B18] Hearne BJ , Teare MD, Butt M, Donaldson L. Comparison of Nottingham Prognostic Index and Adjuvant Online prognostic tools in young women with breast cancer: review of a single-institution experience. BMJ Open 2015;5:e005576. 10.1136/bmjopen-2014-005576 PMC4316437 25628047

[hoae015-B19] Hemingway H , Croft P, Perel P, Hayden JA, Abrams K, Timmis A, Briggs A, Udumyan R, Moons KGM, Steyerberg EW et al; PROGRESS Group. Prognosis research strategy (PROGRESS) 1: a framework for researching clinical outcomes. BMJ 2013;346:e5595. 23386360 10.1136/bmj.e5595 PMC3565687

[hoae015-B20] Hughes EG , King C, Wood EC. A prospective study of prognostic factors in in vitro fertilization and embryo transfer. Fertil Steril 1989;51:838–844. 2707460 10.1016/s0015-0282(16)60676-3

[hoae015-B21] Hunault CC , Habbema JDF, Eijkemans MJC, Collins JA, Evers JLH, Te Velde ER. Two new prediction rules for spontaneous pregnancy leading to live birth among subfertile couples, based on the synthesis of three previous models. Hum Reprod 2004;19:2019–2026. 15192070 10.1093/humrep/deh365

[hoae015-B22] Leushuis E , Steeg JWvd, Steures P, Bossuyt PMM, Eijkemans MJC, Veen FVD, Mol BWJ, Hompes PGA. Prediction models in reproductive medicine: a critical appraisal. †. Hum Reprod Update 2009;15:537–552. 19435779 10.1093/humupd/dmp013

[hoae015-B23] Loendersloot LLv , Wely MV, Repping S, Veen FVD, Bossuyt PMM. Templeton prediction model underestimates IVF success in an external validation. Reprod Biomed Online 2011;22:597–602. 21493154 10.1016/j.rbmo.2011.02.012

[hoae015-B24] Mazzilli R , Vaiarelli A, Dovere L, Cimadomo D, Ubaldi N, Ferrero S, Rienzi L, Lombardo F, Lenzi A, Tournaye H. Severe male factor in in vitro fertilization: definition, prevalence, and treatment. An update. Asian J Androl 2022;24:125. 34259196 10.4103/aja.aja_53_21 PMC8887096

[hoae015-B25] McLernon DJ , Lee AJ, Maheshwari A, van Eekelen R, van Geloven N, Putter H, Eijkemans MJ, van der Steeg JW, van der Veen F, Steyerberg EW et al Predicting the chances of having a baby with or without treatment at different time points in couples with unexplained subfertility. Hum Reprod 2019;34:1126–1138. 31119290 10.1093/humrep/dez049

[hoae015-B26] McLernon DJ , Steyerberg EW, Velde ER, Te Lee AJ, Bhattacharya S. Predicting the chances of a live birth after one or more complete cycles of in vitro fertilisation: population based study of linked cycle data from 113 873 women. BMJ 2016;355:i5735. 27852632 10.1136/bmj.i5735 PMC5112178

[hoae015-B17] National Institute for Health and Care Excellence. Fertility problems: assessment and treatment. 2013; Clinical guideline [CG156]. https://www.nice.org.uk/guidance/cg156 (26 March 2024, date last accessed). 31804780

[hoae015-B28] Nayudu PL , Gook DA, Hepworth G, Lopata A, Johnston WIH. Prediction of outcome in human in vitro fertilization based on follicular and stimulation response variables. Fertil Steril 1989;51:117–125. 2910705 10.1016/s0015-0282(16)60439-9

[hoae015-B29] Nelson SM , Lawlor DA. Predicting live birth, preterm delivery, and low birth weight in infants born from in vitro fertilisation: a prospective study of 144,018 treatment cycles. PLoS Med 2011;8:e1000386. 21245905 10.1371/journal.pmed.1000386 PMC3014925

[hoae015-B30] Pandey S , Shetty A, Hamilton M, Bhattacharya S, Maheshwari A. Obstetric and perinatal outcomes in singleton pregnancies resulting from IVF/ICSI: a systematic review and meta-analysis. Hum Reprod Update 2012;18:485–503. 22611174 10.1093/humupd/dms018

[hoae015-B31] Penzias A , Azziz R, Bendikson K, Cedars M, Falcone T, Hansen K, Hill M, Jindal S, Kalra S, Mersereau J et al Fertility evaluation of infertile women: a committee opinion. Fertil Steril 2021;116:1255–1265. 34607703 10.1016/j.fertnstert.2021.08.038

[hoae015-B32] Pham CT , Karnon JD, Norman RJ, Mol BW. Cost-effectiveness modelling of IVF in couples with unexplained infertility. Reprod Biomed Online 2018;37:555–563. 30361048 10.1016/j.rbmo.2018.08.024

[hoae015-B33] Quaas A , Dokras A. Diagnosis and treatment of unexplained infertility. Rev Obstet Gynecol 2008;1:69–76. 18769664 PMC2505167

[hoae015-B34] Raperport C , Desai J, Qureshi D, Rustin E, Balaji A, Chronopoulou E, Homburg R, Khan KS, Bhide P. The definition of unexplained infertility: a systematic review. BJOG 2023. doi:10.1111/1471-0528.17697. 37957032

[hoae015-B35] Romualdi D , Ata B, Bhattacharya S, Bosch E, Costello M, Gersak K, Homburg R, Mincheva M, Norman RJ, Piltonen T et al; Guideline Group on Unexplained Infertility. Evidence-based guideline: unexplained infertility. Hum Reprod 2023;38:1881–1890. 37599566 10.1093/humrep/dead150 PMC10546081

[hoae015-B36] Sharlip ID , Jarow JP, Belker AM, Lipshultz LI, Sigman M, Thomas AJ, Schlegel PN, Howards SS, Nehra A, Damewood MD et al Best practice policies for male infertility. Fertil Steril 2002;77:873–882. 12009338 10.1016/s0015-0282(02)03105-9

[hoae015-B37] Smeenk JM , Stolwijk AM, Kremer JA, Braat DD. External validation of the Templeton model for predicting success after IVF. Hum Reprod 2000;15:1065–1068. 10783353 10.1093/humrep/15.5.1065

[hoae015-B38] Song Z , Li W, O’Leary S, Roberts B, Alvino H, Tremellen K, Gadalla MA, Wang R, Mol BW. Can the use of diagnostic and prognostic categorisation tailor the need for assisted reproductive technology in infertile couples? Aust N Z J Obstet Gynaecol 2021;61:297–303. 33135775 10.1111/ajo.13273

[hoae015-B39] Souter I , Sun F, Zhang H, Diamond MP, Legro RS, Wild RA, Hansen KR, Santoro N; Eunice Kennedy Schriver National Institute of Child Health and Human Development Reproductive Medicine Network. A personalized medicine approach to ovulation induction/ovarian stimulation: development of a predictive model and online calculator from level-I evidence. Fertil Steril 2022;117:408–418. 35125179 10.1016/j.fertnstert.2021.10.024 PMC8985501

[hoae015-B40] Steures P , Steeg JWvd, Hompes PG, Habbema JDF, Eijkemans MJ, Broekmans FJ, Verhoeve HR, Bossuyt PM, Veen FVD, Mol BW; Collaborative Effort on the Clinical Evaluation in Reproductive Medicine. Intrauterine insemination with controlled ovarian hyperstimulation versus expectant management for couples with unexplained subfertility and an intermediate prognosis: a randomised clinical trial. Lancet 2006;368:216–221. 16844491 10.1016/S0140-6736(06)69042-9

[hoae015-B41] Steures P , van der Steeg JW, Mol BWJ, Eijkemans MJC, van der Veen F, Habbema JDF, Hompes PGA, Bossuyt PMM, Verhoeve HR, van Kasteren YM et al; CECERM (Collaborative Effort in Clinical Evaluation in Reproductive Medicine). Prediction of an ongoing pregnancy after intrauterine insemination. Fertil Steril 2004;82:45–51. 15236988 10.1016/j.fertnstert.2003.12.028

[hoae015-B42] Stolwijk AM , Zielhuis GA, Hamilton CJ, Straatman H, Hollanders JM, Goverde HJ, van Dop PA, Verbeek AL. Prognostic models for the probability of achieving an ongoing pregnancy after in-vitro fertilization and the importance of testing their predictive value. Hum Reprod 1996;11:2298–2303. 8943544 10.1093/oxfordjournals.humrep.a019092

[hoae015-B27] Teede HJ , Misso M, Costello M, Dokras A, Laven J, Moran L, Piltonen T, Norman R; International PCOS Network. Recommendations from the international evidence-based guideline for the assessment and management of polycystic ovary syndrome. *Fertil Steril*. 2018;**110**:364–379. 10.1016/j.fertnstert.2018.05.004 PMC6939856 30033227

[hoae015-B43] Templeton A , Morris JK, Parslow W. Factors that affect outcome of in-vitro fertilisation treatment. Lancet 1996;348:1402–1406. 8937279 10.1016/S0140-6736(96)05291-9

[hoae015-B44] Thurston L , Abbara A, Dhillo WS. Investigation and management of subfertility. J Clin Pathol 2019;72:579–587. 31296604 10.1136/jclinpath-2018-205579

[hoae015-B45] Tjon-Kon-Fat RI , Bensdorp AJ, Bossuyt PMM, Koks C, Oosterhuis GJE, Hoek A, Hompes P, Broekmans FJ, Verhoeve HR, de Bruin JP et al Is IVF—served two different ways—more cost-effective than IUI with controlled ovarian hyperstimulation? Hum Reprod 2015;30:2331–2339. 26269539 10.1093/humrep/dev193

[hoae015-B46] Tomlinson MJ , Amissah-Arthur JB, Thompson KA, Kasraie JL, Bentick B. Infertility: prognostic indicators for intrauterine insemination (IUI): statistical model for IUI success. Hum Reprod 1996;11:1892–1896. 8921060 10.1093/oxfordjournals.humrep.a019513

[hoae015-B47] van der Steeg JWvd , Steures P, Eijkemans MJC, Habbema JDF, Hompes PGA, Broekmans FJ, Dessel HV, Bossuyt PMM, Veen FVD, Mol BWJ; CECERM Study Group (Collaborative Effort for Clinical Evaluation in Reproductive Medicine). Pregnancy is predictable: a large-scale prospective external validation of the prediction of spontaneous pregnancy in subfertile couples. Hum Reprod 2007;22:536–542. 16997935 10.1093/humrep/del378

[hoae015-B48] van Eekelen R , Eijkemans MJ, Mochtar M, Mol F, Mol BW, Groen H, van Wely M. Cost-effectiveness of medically assisted reproduction or expectant management for unexplained subfertility: when to start treatment? Hum Reprod 2020;35:2037–2046. 32876323 10.1093/humrep/deaa158 PMC7550266

[hoae015-B49] van Eekelen R , Scholten I, Tjon-Kon-Fat RI, Steeg JWvd, Steures P, Hompes P, Wely MV, Veen FVD, Mol BW, Eijkemans MJ et al Natural conception: repeated predictions over time. Hum Reprod 2017;32:346–353. 27993999 10.1093/humrep/dew309

[hoae015-B50] Wang R , Danhof NA, Tjon-Kon-Fat RI, Eijkemans MJ, Bossuyt PM, Mochtar MH, Veen FVD, Bhattacharya S, Mol BWJ, Wely M et al; Cochrane Gynaecology and Fertility Group. Interventions for unexplained infertility: a systematic review and network meta-analysis. Cochrane Database Syst Rev2019a;2019:CD012692. 10.1002/14651858.CD012692.pub2 PMC6727181 31486548

[hoae015-B51] Wang R , Eekelen RV, Mochtar MH, Mol F, Wely MV. Treatment strategies for unexplained infertility. Semin Reprod Med 2020;38:48–54. 33124018 10.1055/s-0040-1719074

[hoae015-B52] Wang R , Welie NV, Rijswijk JV, Johnson NP, Norman RJ, Dreyer K, Mijatovic V, Mol BW. Effectiveness on fertility outcome of tubal flushing with different contrast media: systematic review and network meta‐analysis. Ultrasound Obstet Gynecol2019b;54:172–181. 30740799 10.1002/uog.20238

[hoae015-B53] Wessel JA , Mochtar MH, Besselink DE, Betjes H, Bruin JPd, Cantineau AEP, Groenewoud ER, Hooker AB, Lambalk CB, Kwee J et al Expectant management versus IUI in unexplained subfertility and a poor pregnancy prognosis (EXIUI study): a randomized controlled trial. Hum Reprod 2022;37:2808–2816. 36331493 10.1093/humrep/deac236 PMC9712943

[hoae015-B55] Zhang J , Lan W, Wang Y, Chen K, Zhang G, Yang W, Chen H, Xu W, Ma J, Qin W et al Ethiodized poppyseed oil-based contrast medium is superior to water-based contrast medium during hysterosalpingography regarding image quality improvement and fertility enhancement: a multicentric, randomized and controlled trial. EClinicalMedicine 2022;46:101363. 35399811 10.1016/j.eclinm.2022.101363 PMC8987810

